# *MiR-21* in extracellular vesicles contributes to the growth of fertilized eggs and embryo development in mice

**DOI:** 10.1042/BSR20180036

**Published:** 2018-08-31

**Authors:** Chao Lv, Wen-Xian Yu, Yan Wang, Da-Jing Yi, Ming-Hua Zeng, Hong-Mei Xiao

**Affiliations:** Institute of Reproduction and Stem Cell Engineering, School of Basic Medical Science, Central South University, Changsha 410000, P.R. China

**Keywords:** Extracellular vesicles, Embryo development, Embryo implantation, Fertilized eggs, MicroRNA-21, Microinjection

## Abstract

Human preimplantation embryo development is susceptible to high rates of early embryo wastage. We determined the *miR-21* expression of extracellular vesicles (EVs) in fertilized eggs and embryos of varying stages and their response to *miR-21* microinjection. Sexually mature female and male mice were mated. Next, the expression and immunohistochemistry intensity of surface markers (CD9 and CD63) of EVs were detected in pregnant and non-pregnant mice. Exosomes were co-cultured with embryos for detection of blastocyst formation rate, and embryo apoptosis. Moreover, the expressions of Bcl-2 associated X protein (Bax), B cell lymphoma 2 (Bcl-2), and octamer-binding transcription factor-4 (Oct4) were determined. Finally, we detected *miR-21* expression in EVs of uterus in pregnant mice, in embryos after embryo implantation and after embryo co-cultured with exosomes in uterine luminal fluid. *MiR-21* was up-regulated in EVs of uterus, and higher immunohistochemistry intensity of CD9 and CD63, suggesting more EVs secreted in uterine luminal fluid in pregnant mice. After microinjection, *miR-21* inhibitor suppresses embryo development of mice. Moreover, embryos co-cultured with exosomes display higher blastocyst formation rate, reduced apoptotic rate of embryos in pregnant mice. In addition, *miR-21* was down-regulated with the development of embryos after embryo implantation, while *miR-21* expression in embryos was up-regulated by exosomes in uterine luminal fluid in the pregnant mice. Increased *miR-21* expression in EVs of uterus and increased *miR-21* expression after implantation, which indicate the key role in the growth of fertilized eggs and embryo development in mice.

## Introduction

Fertilization, implantation, and embryo development in the early stages are complicated processes that are highly dependent on communication between cells and tissues [1]. Sperm obtains the ability for fertilizing an oocyte gradually during the transition through the epididymis, the interaction with the seminal fluid, passage in the vagina, connection with the epithelium of the oviduct, and fusion with the oocyte [2]. After the growing embryo moves into the uterus, a successful implantation is conducted by the occurrence of apposition, subsequent adherence of the blastocyst to the endometrial luminal epithelium, and the following endometrial invasion [3]. Extracellular vesicles (EVs) are membrane-bound vesicles, which play a key role in intercellular communication by carrying and transferring regulatory molecules, for example miRNAs (miRs) and proteins, acting as vehicles for transportation between cells and tissues [4]. Composition of the EV cargo is complicated, containing a great number of proteins, lipids, DNA, and many small RNA species like miRs [5,6], which are believed to regulate temporal and spatial gene expression by 30–70% [7]. Though we still know little about the functioning patterns and target genes of most miRs, it is recently reported that miRNAs are involved in the processes of embryogenesis and the development of embryos along with the cell proliferation, pluripotency, differentiation, organogenesis, apoptosis, and growth [8].

With the increasing knowledge about miR mechanisms together with the participation in many processes of post-transcriptional regulation, miRs are notable candidates which control the maternal transcripts when in early embryos [9]. Complete development of embryos relates to the proliferation and differentiation of embryonic cells at the early stage, therefore miRs not only play an important role in somite formation, but also are involved deeply in regulating the complete development of embryos [10]. In 2004, several regulatory regions of miR-21 were discovered, since then overexpression of miR-21 has been shown in a variety of pathological conditions containing most kinds of cancers [11]. The first indication of the aberrant expression of miR-21 derived from the miR profiling of human glioblastoma, the most fatal brain tumor of glial origin [12]. Researchers in many kinds of fields such as development, oncology, stem cell biology, and ageing have paid attention to miR-21, and the high expression of miR-21 is found in cancer cells, pathological cell growth, or cell stress [13]. In our study, we made our points on further investigation for the effects of miR-21 expression in EVs on the growth of fertilized eggs and embryo development.

## Materials and methods

### Ethics statement

The experiments were conducted in accordance with the Guide for the Care and Use of Laboratory Animals published by the National Institutes of Health [14] and followed by the approval of the Animal Committee of Central South University.

### Experimental animals

Sexually mature female C57B6/J mice aged 4–5 weeks (*n*=134) and male C57B6/J mice aged 11–15 weeks (*n*=134) were purchased from Hunan SJA Laboratory Animal Co., Ltd., (Changsha, Hunan, China). Mice were normally fed for 3 days, and then intraperitoneally injected with 10 IU pregnant mare serum gonadotropin (PMSG) (Ningbo Second Hormone Factory, Ningbo, Zhejiang, China) at 5 p.m. on the fourth day, followed by injecting with 10 IU human chorionic gonadotropin (HCG) (Ningbo Second Hormone Factory, Ningbo, Zhejiang, China) after 48 h. Following this, these female and male mice were mated at a ratio of 1:1 overnight. The pregnancy conditions were observed next morning. Successful mating was confirmed by the detection of a vaginal plug. And 2-cell stage was determined from 22 to 24 h after injection of HCG according to the development time of mouse’s fertilized eggs, and at this time 30 pregnant female mice were killed. Then, bilateral fallopian tubes were removed from the abdomens carefully. After that, the fimbriae of fallopian tubes were injected with PBS solution under a dissecting microscope (1.9 mM monobasic potassium phosphate, 8.1 mM dibasic potassium phosphate, 75 mM sodium chloride, pH = 7.4). Then, the fertilized eggs were flushed out and placed in M2 medium with 12 h of pre-equilibration. After microinjection, the fertilized eggs were washed twice by M16 medium with preheating, again resuspending in M16 medium with preheating and maintained in culture dish under the coverage of mineral oil. Finally, the fertilized eggs were cultured in the incubator with 5% CO_2_ at 37°C with saturated humidity.

### Cell grouping and cytoplasmic microinjection

The mimic (AUCGAAUAGUCUGACUACAACUAAAAAA) for mature fragment of miR-21 (Genbank number: NR_029738.1) was synthetized by Sangon Biotech Co., Ltd. (Shanghai, China), and miR-21 inhibitor was purchased from Qiagen (219300, Qiagen, Valencia, CA, U.S.A.). RNA was dissolved and the concentration was adjusted to 25 mmol/l by trace element (TE) solution (10 mmol/l Tris/HCl, 1 mmol/l EDTA, pH = 8.0). *In vitro* fertilized eggs in 2-cell stage were divided into the blank group (without microinjection), experimental group (microinjection with miR-21 or miR-21 inhibitor), and negative control (NC) group (microinjection with TE solution). During the microinjection into the cytoplasm, the cell surface of fertilized eggs in 2-cell stage was found under an inverted microscope of low magnification. The cytoplasm was slowly injected with 10 pl TE solution or miR-21 and miR-21 inhibitor solution dissolved in TE solution, carefully and precisely by a microinjector. The fertilized eggs were cultured and observed for the development at 12, 24, and 36 h under an inverted microscope.

### Isolation of EVs and immunohistochemical staining

The pregnant female mice and the non-pregnant female mice were classified as the pregnancy group and the non-pregnancy group, respectively. According to *Manipulating the mouse embryo: a laboratory manual* [15], the period from 76 to 78 h after HCG injection was speculated as the early stage of blastocyst. At this time, eight pregnant female mice and eight non-pregnant female mice were killed. Uteruses were removed from abdomens of mice carefully, and uterine luminal fluid was flushed out with 1 ml PBS solution. Cell debris was removed by centrifugation at 21000 ***g*** for 15 min at 4°C, and the supernatant was filtered by 0.22-µm nylon membrane. The EVs were extracted by standard method of total EV extraction kit (BD Biosciences, Franklin Lakes, NJ, U.S.A.). Exosome TEM, SEM, and particle-size analysis were conducted by Shanghai XP Biomed Co., Ltd., (Shanghai, China).

Immunohistochemical staining was used to determine the expressions of such surface markers as CD9 and CD63 on EVs. The endometrial sections of pregnant and non-pregnant mice were taken out, fixed with 4% formaldehyde, embedded with paraffin, and conventionally dewaxed to water. The activity of endoperoxidase was blocked by 3% hydrogen peroxide for 1 h. Then the specimens were washed three times with PBS solution (2 min per time), and added with a drop of rabbit anti-mouse CD9 (1:100, product ID: ab92726, Abcam PLC, Cambridge, U.K.) and a drop of rabbit anti-mouse CD63 (1:100, product ID: GTX37555, GeneTex, Irvine, CA, U.S.A.), respectively. After 1 h of incubation at 37°C, the specimens were washed three times with PBS solution (2 min per time) and added with secondary antibody EnVision (Zhongshan Goldenbridge Biotechnology Co., Ltd., Beijing, China). After incubation at room temperature for 30 min, the specimens were again washed three times with PBS solution (2 min per time), and colored with DAB chromogenic reagent (Zhongshan Goldenbridge Biotechnology Co., Ltd., Beijing, China). The reaction was terminated by running water when yellow precipitate appeared. After coloration, nucleuses were re-stained with Hematoxylin. The sections were dehydrated in conventional gradient alcohol after bluing, permeabilized in xylene and then mounted with neutral balsam. The results of immunohistochemical staining were scored by three readers. Scores of staining were 1–4 points (low coloration, medium coloration, high coloration, and extremely high coloration).

### Western blot analysis

The tissue samples (30 mg) were taken out and ground into fine powder in liquid nitrogen. Next, the samples were added with protein lysate solution and protease inhibitor (A37989, Thermo Fisher Scientific, CA, U.S.A.), and placed on the ice for 20 min. The lysate was centrifuged at the rate of 12000 rpm for 20 min for obtaining supernatant. The concentration of total proteins was measured using BCA kit (23227, Thermo Fisher Scientific, CA, U.S.A.). After detection of protein concentration of extracted exosome, the 25 μg protein was used for experiment. The protein (50 μg) was extracted and dissolved in 2× SDS loading buffer and boiled at 100°C for 5 min. Next, the samples were treated with SDS/PAGE (12% gel), and transferred on to PVDF membrane using the wet transfer method. Then, the membranes were blocked in 5% dried skimmed milk for 1 h at room temperature, followed by washing with TBS for 15 min. Subsequently, the PVDF membrane was incubated with the diluted primary antibody, and rinsed with TBS and Tween 20 (TBST) three times, and incubated with the secondary antibody goat anti-rabbit labeled with horseradish peroxidase (HRP) (1:5000) for 1 h. After rinsing with TBST three times (15 min per time), the membrane was developed by electrochemiluminescence (ECL), exposed by X-ray, and photographed. The experiment was repeated three times.

### Co-culture of exosome and embryo

The obtained fertilized eggs in the 2-cell stage *in vitro* were classified into the control (normal culture embryos), non-pregnancy (exosome added with 5 μl uterine luminal fluid of non-pregnant mice), and pregnancy (exosome added with 5 μl uterine luminal fluid of pregnant mice) groups. The exosome and embryo were cultured into the blastocyst stage. Next, the blastocyst formation rate was recorded, and the embryo apoptosis rate and blastocyst inner cell mass/trophectoderm (ICM/TE) ratio were detected.

### Embryo collection

According to *Manipulating the mouse embryo: a laboratory manual* [16], 18, 22–24, 40–42, 54-56, 62–64, 69–71, 76–78, and 82–86 h after injection of HCG were speculated as the period of mononuclear cells, 2-cell stage, 4-cell stage, 8-cell stage, 16-cell stage, morula and mature blastocyst of fertilized eggs. After injection of HCG for 142–156 h, embryo implantation happened. After 22–24, 40–42, 54–56, 62–64, 69–71, and 82–86 h of HCG injection, eight pregnant female mice were killed respectively. Then the bilateral fallopian tubes were removed from the abdomens carefully and 1 ml PBS solution was inserted by a pipette through one side of the fallopian tubes. After that the fertilized eggs in 2-cell stage, 4-cell stage, 8-cell stage, 16-cell stage together with morula and blastocyst were obtained for further experiments. The day of plug detection was considered as the first day of pregnancy, and embryo implantation happened on the 6th day of pregnancy. Five embryos were collected on the 6th, 9th, 12th, 15th, and 18th days of pregnancy, respectively. Reverse-transcription quantitative PCR (RT-qPCR) was adopted to determine the miR-21 expression in these embryos.

### RT-qPCR

Total RNA of EVs and mouse embryonic cells were extracted by an RNA extraction kit (Tiandz Gene Technology Co., Ltd., Beijing, China). The cDNA was produced through reverse transcription using RT-qPCR kit (Bioer Technology Co., Ltd., Hangzhou, Zhejiang, China). Then, RT-qPCR was used for detecting the target mRNA expression in specimens. The primer sequences were as follows. Forward primer of miR-21 was 5′-ATGGTTCGTGGTAGCTTATCAGACTGA-3′ and reverse primer was 5′-GCAGGGTCCGAGGTATTC-3′. U6 RNA was regarded as internal reference. Forward primer of U6 RNA was 5′-GCTTCGGCAGCACATACTAAAAT-3′ and reverse primer was 5′-CGCTTCACGAATTTGCGTGTCAT-3′. Forward primer of Bcl-2 associated X protein (Bax) was 5′-TCCCACATAACTCCCTCGACA-3′ and reverse primer was 5′-GGCGAAGCCAGCGAGAAGTCCC-3′. Forward primer of B cell lymphoma 2 (Bcl-2) was 5′-GACAGAAGATCATGCCGTCC-3′ and reverse primer was 5′-CTTTGATGTCACGCACGATTTC-3′. Forward primer of octamer-binding transcription factor-4 (Oct4) was 5′-GAGAGGTCCAACGGAGAGTG-3′ and reverse primer was 5′-ACATGAGGAGCCAGGGTAAG-3′. Glyceraldehyde-3-phosphate dehydrogenase (GAPDH) was regarded as the internal reference. Forward primer of GAPDH was 5′-TTCACCACCATGGAGAAGGC-3′ and reverse primer was 5′-GGCATGGACTGTGGTCATGA-3′. Quantitative PCR instrument (7500, Applied Biosystems Inc., Foster City, CA, U.S.A.) was used for RT-qPCR, and RT-PCR mixture was purchased from Bio-Rad (Hercules, CA, U.S.A.). The conditions of PCR were predenaturation at 94°C for 5 min, denaturation at 94°C for 30 s, annealing at 58°C for 30s, and extension at 72°C for 20 s, with 40 cycles. The difference in mRNA expression between the experimental group and control group was expressed by *n*=2^−ΔΔ*C*^_t_ [17].

### Exosome absorption and inhibition

The obtained fertilized eggs in the 2-celled stage *in vitro* were classified into the blank (the fertilized eggs without exosomes and inhibitors), exosome (the fertilized eggs added with exosomes of uterine luminal fluid of pregnant mice), and exosome + inhibitor (the fertilized eggs added with exosomes of uterine luminal fluid of pregnant mice, and exosome absorption inhibitor Pitstop 2 (ab120687, Abcam Inc., Cambridge, MA, U.S.A.) and genistein (S1342, Selleck Chemicals, TX, U.S.A.) groups. The embryos in the 2-cell, 4-cell, and 8-cell stages were selected for RT-qPCR.

### Statistical analysis

SPSS 21.0 software (IBM Corp, Armonk, NY, U.S.A.) was applied for data analysis. The measurement data were expressed as mean ± S.D. and normality test was performed. Comparisons of measurement data were conducted by *t* test, and the Chi-square test was used for the comparisons of enumeration data. *P*<0.05 was considered to be statistically significant.

## Results

### More EVs secreted in uterine luminal fluid in pregnant mice

**Figure 1:**
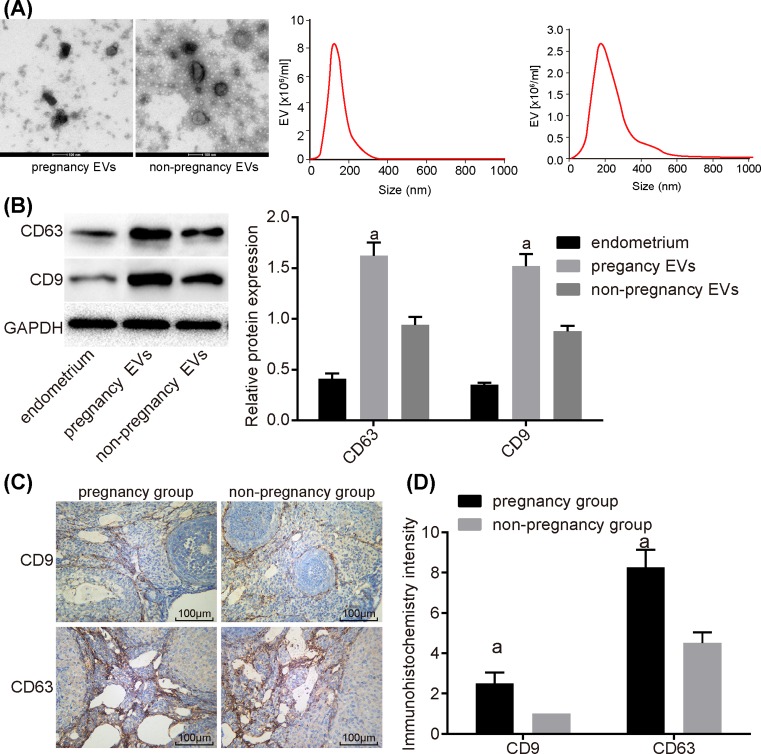
Western blot analysis and immunohistochemical staining show more EVs secreted in uterine luminal fluid in pregnant mice (**A**) Results of electron microscope and particle-size analysis in the endometrium, pregnancy EVs, and non-pregnancy EVs, scale bars = 100 nm. (**B**) Western blot analysis was adopted to detect surface markers CD9 and CD63. (**C**) Immunohistochemical staining of CD9 and CD63. (**D**) The immunohistochemistry intensity of CD9 and CD63 using immunohistochemical staining; ^a^, *P*<0.05, compared with the non-pregnancy group; the measurement data were expressed as mean ± S.D., and single-factor ANOVA was used to analyze the data; *n*=8; scale bars = 100 μm.

Uterine luminal fluid of pregnant mice and non-pregnant mice was separated and the EVs in uterine luminal fluid were extracted. Electron microscope and particle-size analysis showed that the diameter of exosomes was between 60 and 150 nm, and Western blot analysis was performed to detect the markers of EVs, CD9 and CD63. The results revealed that the expressions of CD9 and CD63 were higher in the pregnancy group than those in the non-pregnancy group, indicating that pregnant mice secreted more EVs. The immunohistochemical staining was used to analyze the expressions of such surface markers as CD9 and CD63 in EVs. EVs were observed both in the pregnancy group and the non-pregnancy group. Compared with non-pregnant mice, the staining intensity of CD9 and CD63 in pregnant mice was higher (*P*<0.05), indicating that there were more EVs generating in the uterine luminal fluid of pregnant mice (Figure 1).

### Increased miR-21 expression is identified in pregnant mice

**Figure 2:**
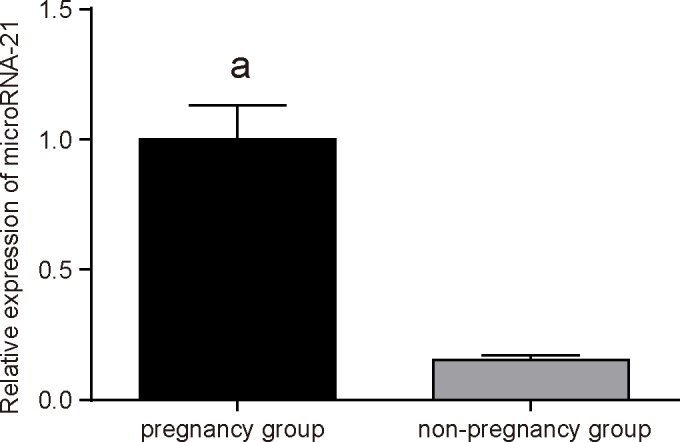
RT-qPCR demonstrates higher expression of miR-21 in EVs of uterus in the pregnancy group ^a^, *P*<0.05, compared with the non-pregnancy group; the measurement data were expressed as mean ± S.D., and *t* test was applied for analysis; *n*=3.

RT-qPCR was used for analyzing the miR-21 expression in EVs of uterus between the pregnancy group and the non-pregnancy group. The result showed that, compared with the non-pregnancy group, miR-21 expression in EVs of uterus was significantly increased in the pregnancy group (*P*<0.05) (Figure 2), which suggests that miR-21 expression was up-regulated in pregnant mice.

### MiR-21 inhibitor suppresses embryo development in mice

**Figure 3:**
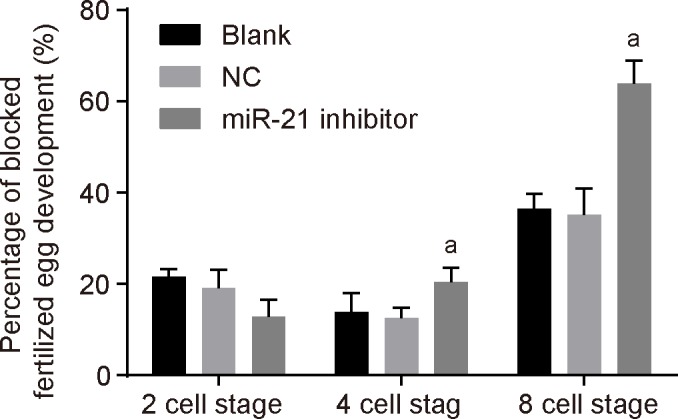
MiR-21 inhibitor suppresses embryo development in mice after microinjection The measurement data were expressed as mean ± S.D., and single-factor ANOVA was used to analyze the data; *n*=3; ^a^, *P*<0.05, compared with the NC group.

The division and the development of fertilized eggs were observed under an inverted microscope amongs the three groups. The percentage of fertilized eggs in 2-cell stage, 4-cell stage, and 8-cell stage, and the percentage of fertilized eggs that was blocked in 2-cell stage were calculated, and the embryos that blocked and died in 2-cell stage, 4-cell stage, and 8-cell stage were regarded as dead embryos. The results indicated that there was no difference in the percentage of fertilized egg development blocked in 2-cell stage, 4-cell stage, and 8-cell stage between the blank group and the NC group (*P*>0.05). Compared with the NC group, miR-21 inhibitor group showed that there was no significant difference in the percentage of fertilized egg development blocked in 2-cell stage (*P*>0.05), while the percentage of fertilized egg development blocked in 4-cell stage and 8-cell stage was remarkably higher (*P*<0.05) (Figure 3). After injection of miR-21 inhibitor, there was no significant difference in arrest of embryo development between the blank and NC groups, and the percentage of arrest of embryo development in the miR-21 inhibitor group was higher than that in the blank and NC groups (Table 1). These findings suggested that miR-21 inhibitor suppresses embryo development of mice.

**Table 1:** The percentage of arrest of embryo development in each group

Treatment groups	Number of oocytes	2-cell stage	4-cell stage	8-cell stage
Blank	61	18 ± 0.9	16 ± 1.2^1^	10 ± 1.3^1^
NC	72	16 ± 1.2	17 ± 0.7^1^	12 ± 0.5^1^
MiR-21 inhibitor	68	19 ± 0.7	56 ± 3.2^2^	78 ± 2.9^2^

Different numbers (^1,2^) of superscripts in the same column indicate significant difference (*P*≤0.05) between treatment groups.

### Embryos cultured with exosomes display higher blastocyst formation rate, ICM/TE ratio, expressions of Bcl-2 and Oct4, and lower expression of Bax in pregnant mice

**Figure 4:**
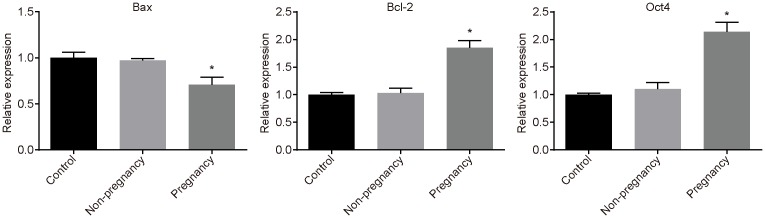
Embryos co-cultured with exosomes display higher blastocyst formation rate and ICM/TE ratio and reduced apoptosis of embryos in pregnant mice The measurement data were expressed as mean ± S.D., and single-factor ANOVA was used to analyze the data; *n*=3; *, *P*<0.05, compared with the control group.

The blastocyst formation rate was used to evaluate the effects of exosomes in uterine luminal fluid on embryo development. As shown in Table 2, compared with the control and non-pregnancy groups, the blastocyst formation rate was obviously higher in the pregnancy group (*P*<0.05). Additionally, the results of RT-qPCR showed that expressions of Bcl-2 and Oct4 were evidently higher than those in the control and non-pregnancy groups, and the Bax expression was significantly lower than that in the control and non-pregnancy groups (all *P*<0.05) (Figure 4).

**Table 2:** Effects of exosome supplementation on development of mouse embryos *in vitro*

Groups	Number of embryos cultured	Number of blastocysts (%)	Apoptotic rate (%)	ICM/TE (%)
Control	315	82 (26.03%)	7.1 ± 2.1	28.6 ± 3.3
Non-pregnancy	302	87 (28.8)	6.8 ± 2.3	29.5 ± 2.9
Pregnancy	311	114 (36.65%)*	2.1 ± 1.3*	38.7 ± 3.9*

*, *P*<0.05, compared with the control and non-pregnancy groups.

### MiR-21 is down-regulated with the development of embryos after embryo implantation

**Figure 5:**
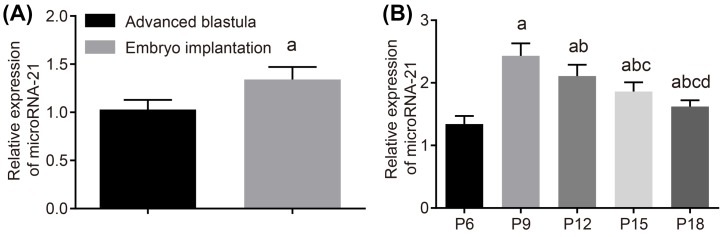
RT-qPCR shows that miR-21 is down-regulated with the development of embryos after embryo implantation (**A**) Expression of miR-21 between advanced blastocyst and embryos; ^a^, *P*<0.05, compared with the blastocyst. (**B**) Expression of miR-21 in the middle and late period of embryo development. The measurement data were expressed as mean ± S.D., and single-factor ANOVA was used to analyze the data; *n*=8; ^a^, *P*<0.05, compared with P6; ^b^, *P<*0.05, compared with P9; ^c^, *P*<0.05, compared with P12; ^d^, compared with P15, *P*<0.05. Abbreviations: P6, 6th day of pregnancy; P9, 9th day of pregnancy; P12, 12th day of pregnancy; P15, 15th day of pregnancy; P18, 18th day of pregnancy.

The embryo implantation occurred on the 6th day of pregnancy, during which the miR-21 expression in the implanted embryos was compared with that in mature blastocysts. The result showed that the miR-21 expression in the embryo after implantation was increased by nearly 20-times than that in the mature blastocyst, suggesting that miR-21 expression was significantly activated by implantation (Figure 5A).

The miR-21 expression of embryos after implantation on the 6th, 9th, 12th, 15th, and 18th days of pregnancy was analyzed and the result showed that miR-21 expression was remarkably activated after implantation. From the 6th day of pregnancy to the 9th day of pregnancy, the miR-21 expression was significantly increased by nearly 20-times again, and the expression reached the peak on the 9th day of pregnancy, then the miR-21 expression was decreased with the development of the embryo (*P*<0.05) (Figure 5B).

All in all, the expression of miR-21 was remarkably increased after the implantation. And relatively high expression of was maintained in the middle and late period of the embryo development, implying that miR-21 might play a significant role in the development of embryos.

### Exosomes in uterine luminal fluid in pregnant mice increases the miR-21 expression in embryos

**Figure 6:**
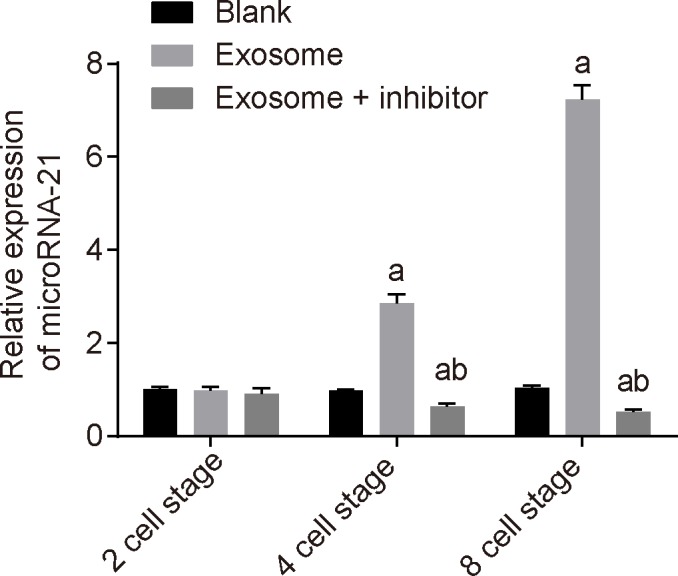
Exosomes in uterine luminal fluid in pregnant mice elevates the miR-21 expression in embryos The measurement data were expressed as mean ± S.D., and single-factor ANOVA was used to analyze the data; *n*=3; ^a^, *P*<0.05, compared with the blank group; ^b^, *P*<0.05, compared with the exosome ± inhibitor group.

The embryo culture fluid was added with absorption inhibitors Pitstop 2, an inhibitor of clathrin-dependent endocytosis, and genistein, an inhibitor of caveolae-dependent endocytosis. The results indicated that compared with the exosome group, the exosome + inhibitor group showed significantly lower miR-21 expression than the blank group (*P*<0.05). The findings suggested that after uptake by embryos, the exosomes in uterine luminal fluid in pregnant mice elevates the miR-21 expression in embryos (Figure 6).

## Discussion

The culture of preimplantation embryos *in vitro* plays a crucial role in human-assisted reproductive technology and animal embryo engineering, while, in mammals, most of the embryos experience a developmental blockage at the early stage, which results in a decrease in the birth rate [18]. Embryo developmental blockage is closely associated with genetic and environmental factors, and apoptosis acts as one of the main causes of embryo death [19]. It was previously demonstrated that miR-21 always has anti-apoptotic effects on many cellular processes [20,21]. The previous studies suggested that miR-21 was involved in embryo implantation [22,23]. Thus, the aim of our research was to investigate the effect of miR-21 expression on the growth of fertilized eggs and the embryo development, trying to find a way to overcome this blocking phenomenon and deal with the problem of high embryo death rate. Finally, all the results in our study indicated that miR-21 positively affect the growth of fertilized eggs and the embryo development under *in vitro* culture.

Initially, we studied the effect of miR-21 on the growth of fertilized eggs, and found that there was no significant difference in percentage of fertilized egg development blocked in 2-cell stage, while the percentage of fertilized egg development blocked in 4-cell stage and 8-cell stage were significantly higher in comparison with the NC group, indicating that miR-21 may plays a key role in promoting the growth of fertilized eggs. Strong evidence has shown that miRs affect cell proliferation, growth, differentiation, and apoptosis by negatively regulating gene expression [24]. Specially, a previous study revealed that miR-21 regulates a variety of cellular and biological processes as a main anti-apoptotic element [25]. It was also previously shown that miR-21 regulates the anti-apoptotic ability of preimplantation embryos [26].

Second, our study showed that compared with the non-pregnancy group, the staining intensity of CD9 and CD63 in the pregnancy group was higher. Similarly, a previous study has revealed positive CD63 protein in pregnant ewes [27]. Moreover, the findings showed that the miR-21 expression in EVs in the pregnancy group was remarkably higher than that in the non-pregnancy group. For these reasons, we inferred that miR-21 plays an important role in fertilization. A previous study provides evidence that miR-21 is very important in preimplantation embryo development, and the preimplantation embryo development quality is significantly associated with the expression of apoptotic proteins, which are regulated by miR-21 [28]. In another study, the research data indicated that low expression of miR-21 in the placenta was always linked with poor fetal growth [18].

Additionally, the data in present study implied that embryos cultured with exosomes display higher blastocyst formation rate, expressions of Bcl-2 and Oct4, lower expression of Bax, and apoptotic rate of embryos in pregnant mice. Qu et al. [29] have demonstrated that embryos produced *in vitro* from lots of mammalian species often reveal fewer blastocyst formation, higher apoptosis index, while supplementation of exosomes in embryos could elevate blastocyst formation rate, and expression of Oct-4. It is reported that increased expression of pro-apoptosis protein Bax and reduced expression of anti-apoptotic protein bcl-2 are significant markers of apoptosis [30]. Moreover, miR-21 overexpression is identified to induce cell apoptosis [31]. A considerable amount of literature has been published on effects of miR-21 on inhibiting apoptosis [32–34]. All these evidence are consistent with our findings.

Furthermore, as shown in our results, miR-21 expression was remarkably increased after implantation. Besides, the miR-21 expression kept relatively high in the middle and late period of the embryo development, suggesting that miR-21 may have effects on the whole process of the embryo development. It is reported that miR-21 is expressed during normal embryogenesis and is strictly regulated in normal development of mice [35]. A former study has already demonstrated that IL-6, which is positively regulated by anti-apoptotic miR-21 expression, plays a role in improving the viability of preimplantation embryos by inducing Stat3 signaling, conforming the anti-apoptotic effect of miR-21 on preimplantation embryos [26]. In addition, a research proved that icariin, a part of the TCM monomer, up-regulates the expression of miR-21 to increase the expression of Bcl-2 and reduce the expression of caspase3 in preimplantation embryos, which displays anti-apoptotic effects on embryo developmental improvement, demonstrating that the developmental quality of preimplantation embryo is closely associated with the expression of apoptosis-related gene that is regulated by miR-21 [36].

In summary, our findings provide evidence that miR-21 have positive effects on the growth of fertilized eggs and the embryo development, which gives a value for the prevention of the high embryo death rate. However, the specific mechanism of miR-21 functioning in the fertilized eggs and the embryos may be influenced by many factors which need further investigation.

## Abbreviations

Bax: Bcl-2 associated X protein
Bcl-2: B cell lymphoma 2
EV: extracellular vesicle
GAPDH: glyceraldehyde-3-phosphate dehydrogenase
HCG: human chorionic gonadotropin
ICM/TE: inner cell mass/trophectoderm
miR: miRNA
NC: negative control
Oct4: octamer-binding transcription factor-4
RT-qPCR: reverse-transcription quantitative PCR
TBST: TBS and Tween 20
TE: trace element
